# Functional examination of lncRNAs in allotetraploid *Gossypium hirsutum*

**DOI:** 10.1186/s12864-021-07771-3

**Published:** 2021-06-13

**Authors:** Luyao Wang, Jin Han, Kening Lu, Menglin Li, Mengtao Gao, Zeyi Cao, Ting Zhao, Xue Chen, Xiaoyuan Tao, Quanjia Chen, Xueying Guan

**Affiliations:** 1 Engineering Research Centre of Cotton, Ministry of Education / College of Agriculture, Xinjiang Agricultural University, 311 Nongda East Road, 830052 Urumqi, China; 2 State Key Laboratory of Crop Genetics and Germplasm Enhancement, Cotton Hybrid R & D Engineering Center (the Ministry of Education), College of Agriculture, Nanjing Agricultural University, 210095 Nanjing, Jiangsu China; 3 College of Agriculture and Biotechnology, Zhejiang University, 210058 Hangzhou, Zhejiang China; 4 Hainan Institute of Zhejiang University, Yazhou Bay Science and Technology City, Building 11, Yonyou Industrial Park, Yazhou District, Hainan Province 572025 Sanya, China

**Keywords:** lncRNA function, Conservation, Abiotic stress

## Abstract

**Background:**

An evolutionary model using diploid and allotetraploid cotton species identified 80 % of non-coding transcripts in allotetraploid cotton as being uniquely activated in comparison with its diploid ancestors. The function of the lncRNAs activated in allotetraploid cotton remain largely unknown.

**Results:**

We employed transcriptome analysis to examine the relationship between the lncRNAs and mRNAs of protein coding genes (PCGs) in cotton leaf tissue under abiotic stresses. LncRNA expression was preferentially associated with that of the flanking PCGs. Selected highly-expressed lncRNA candidates (n = 111) were subjected to a functional screening pilot test in which virus-induced gene silencing was integrated with abiotic stress treatment. From this low-throughput screen, we obtained candidate lncRNAs relating to plant height and tolerance to drought and other abiotic stresses.

**Conclusions:**

Low-throughput screen is an effective method to find functional lncRNA for further study. LncRNAs were more active in abiotic stresses than PCG expression, especially temperature stress. LncRNA XLOC107738 may take a *cis-*regulatory role in response to environmental stimuli. The degree to which lncRNAs are constitutively expressed may impact expression patterns and functions on the individual gene level rather than in genome-wide aggregate.

**Supplementary Information:**

The online version contains supplementary material available at 10.1186/s12864-021-07771-3.

## Background

More than 90 % of all transcripts in eukaryotic genomes cannot be translated into proteins. A large proportion of these transcripts are long non-coding RNAs (lncRNAs) [1]. Despite not being protein-coding, these lncRNAs are also under pressure for natural and human selection during evolution. A comparative analysis of the human and mouse genomes determined that lncRNAs are under positive selection rather than neutral mutation [2]. Some lncRNAs are also known to be affected by positive selection in domesticated species, such as *BRAFP* in mammals [3]; altered expression of lncRNAs during domestication has likewise been identified in rice [4]. Therefore, lncRNAs actively take part in the evolution of species just as coding genes do. In addition, difference species exhibit distinctly different lncRNA profiles ; for example, the mouse and rat genomes share only 2,572 lncRNAs, comprising ~ 12.7 and 11.1 % of their total respective lncRNA profiles [5], and in citrus, no more than 10 % of intergenic lncRNAs in *Atalantia* could be aligned to other citrus species [6]. However, a few functional lncRNAs are reported to be conserved across species; one such is *Xist* in mice and humans [7].

One mechanism by which noncoding RNAs can be functional is presented by the competitive endogenous RNA (ceRNA) hypothesis [3], which proposes different transcripts compete for binding of shared miRNAs. This hypothesis fits pseudogenes such as *BRAFP1* that retain the miRNA targeting sequences of their parental coding genes and exhibit evolutionary conservation across species [8]. In contrast, the majority of lncRNA transcripts are generated from transposon elements and intergenic regions [1, 9].

The roles of lncRNAs in plants are of considerable interest, having emerged as new epigenetic regulators of coding gene expression in biological activities and as specifically affecting plant responses to abiotic stress [10–13]. In particular, lncRNAs are involved in transcriptional gene silencing, gene expression regulation, chromatin structural remodeling, and other epigenetic mechanisms [14, 15]. Two common modes of regulation by lncRNAs involve non-local actions in *trans* and local actions in *cis*, which regulate the expression of adjacent genes [16–18]. For example, *COLDAIR* recruits PRC2 (Polycomb Repressive Complex 2) to regulate *FLOWERING LOCUS C* (*FLC*) in *trans* [19], as*DOG1* inhibits the transcription of *DOG1* on the opposite chain acts in *cis* [20]. Although a number of lncRNAs have been identified, only a few are functionally well characterized. *COOLAIR* is a lncRNA with a conserved secondary structure, which has been proposed evolutionarily conserved across species and cis-regulate the *FLC* [21, 22], *MuLnc1* in mulberry [23], the involvement of cotton lncRNA973 in response to salt stress [24], lncRNA1459 altering tomato fruit ripening [25], and T5120 as a regulator of nitrate response and assimilation in *Arabidopsis* [26]. Though lncRNAs have been identified at the genome level in many plants or conditions [27–30], it remains difficult to characterize functional candidates.

The allotetraploid evolutionary model is an appropriate system to investigate lncRNA function. Within the diploid parent species and the allotetraploid species, non-coding regions can be compared in terms of sequence similarity and syntenic relationships. Furthermore, global genomic comparisons have revealed that lncRNAs burst in the process of polyploidization [31]. An analytical model system applying a comparative genomic method to allotetraploid cotton lncRNAs has been developed [31, 32]. The diploid ancestors of the cotton A and D subgenomes underwent interspecific hybridization and polyploidy to form allotetraploid cotton one to two million years ago [33]. As research regarding the evolutionary genomics, population genetics, and epigenetics of allotetraploid cotton has developed rapidly [34–38], this provides a useful model system in which to further examine the rapid evolution of lncRNAs.

Thus, the functional relationship of lncRNA and protein-coding genes (PCGs) were further analyzed with the applied abiotic stresses in cotton leaves. We continued to use the lncRNA evolution system in cotton to investigate the functional lncRNAs in *G. hirsutum*.

## Results

### The source of lncRNAs activated in *Gossypium hirsutum*

**Fig. 1:**
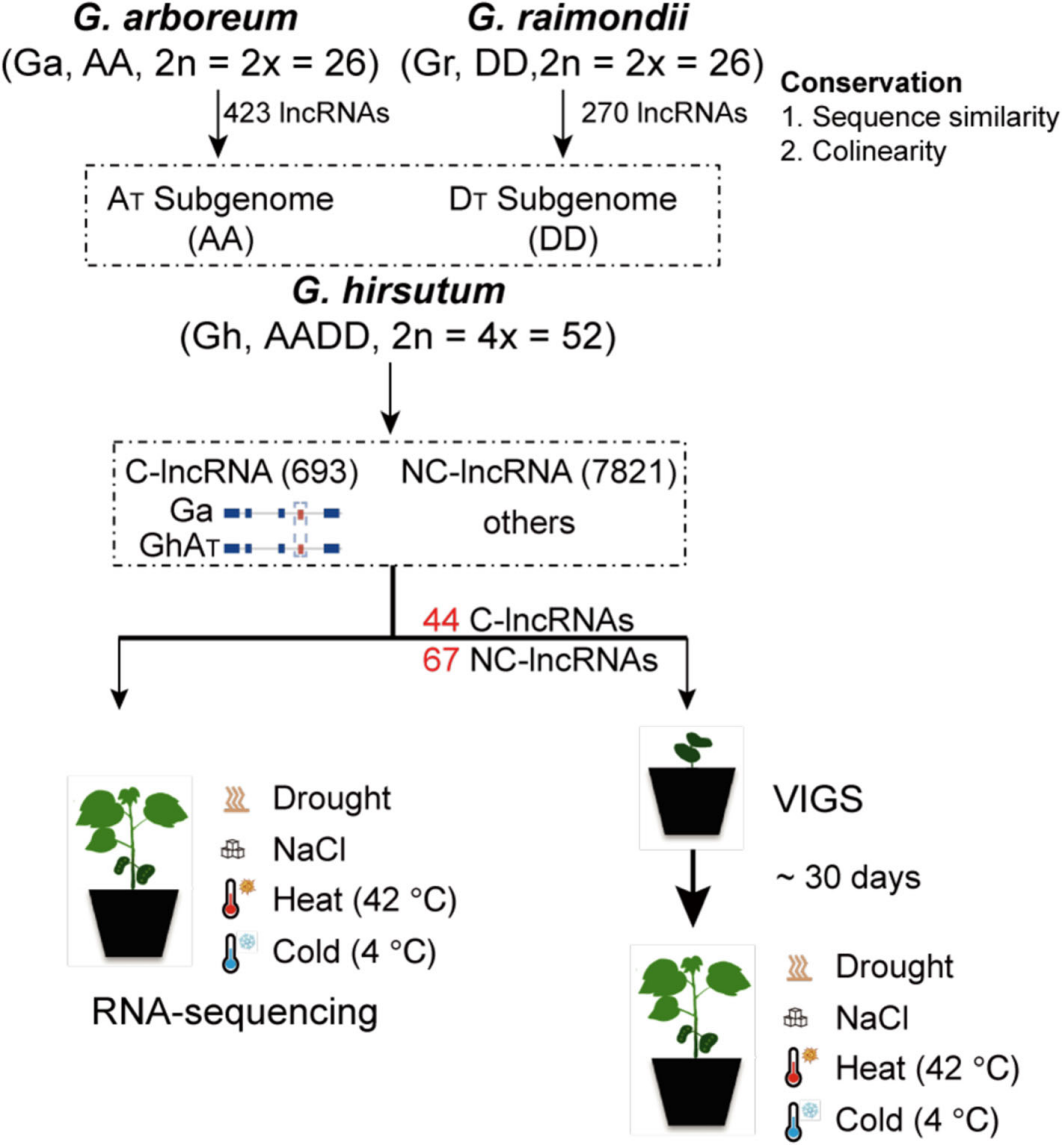
Workflow of functional lncRNA screening in upland cotton (*Gossypium hirsutum*)

The function of a lncRNA may be related to its sequence conservation, location on the chromosome, and transcriptional activity [39]. In our previous study, we respectively identified 4,107, 2,381, and 8,514 lncRNAs in the *G. arboreum* (Ga), *G. raimondii* (Gr), and *G. hirsutum* (Gh) genomes. We then classified conserved lncRNAs (C-lncRNAs) and non-conserved lncRNAs (NC-lncRNAs) in Gh based on (1) the sequence similarity (blastn -evalue 10^^−10^ -max_target_seqs 1), (2) collinearity on the chromosome (MCscanX -b 2, -s 5) of the lncRNA for downstream functional analysis. A total of 693 lncRNAs in Gh were retained from Ga or Gr, and 7821 were activated in Gh (Fig. 1, Table S1 and S2). That C-lncRNAs comprised only about 10 % of all identified lncRNAs is in agreement with the common observation that most lncRNAs are unique to each species [32, 40].

### Expression pattern analysis of lncRNAs under abiotic stresses

**Fig. 2:**
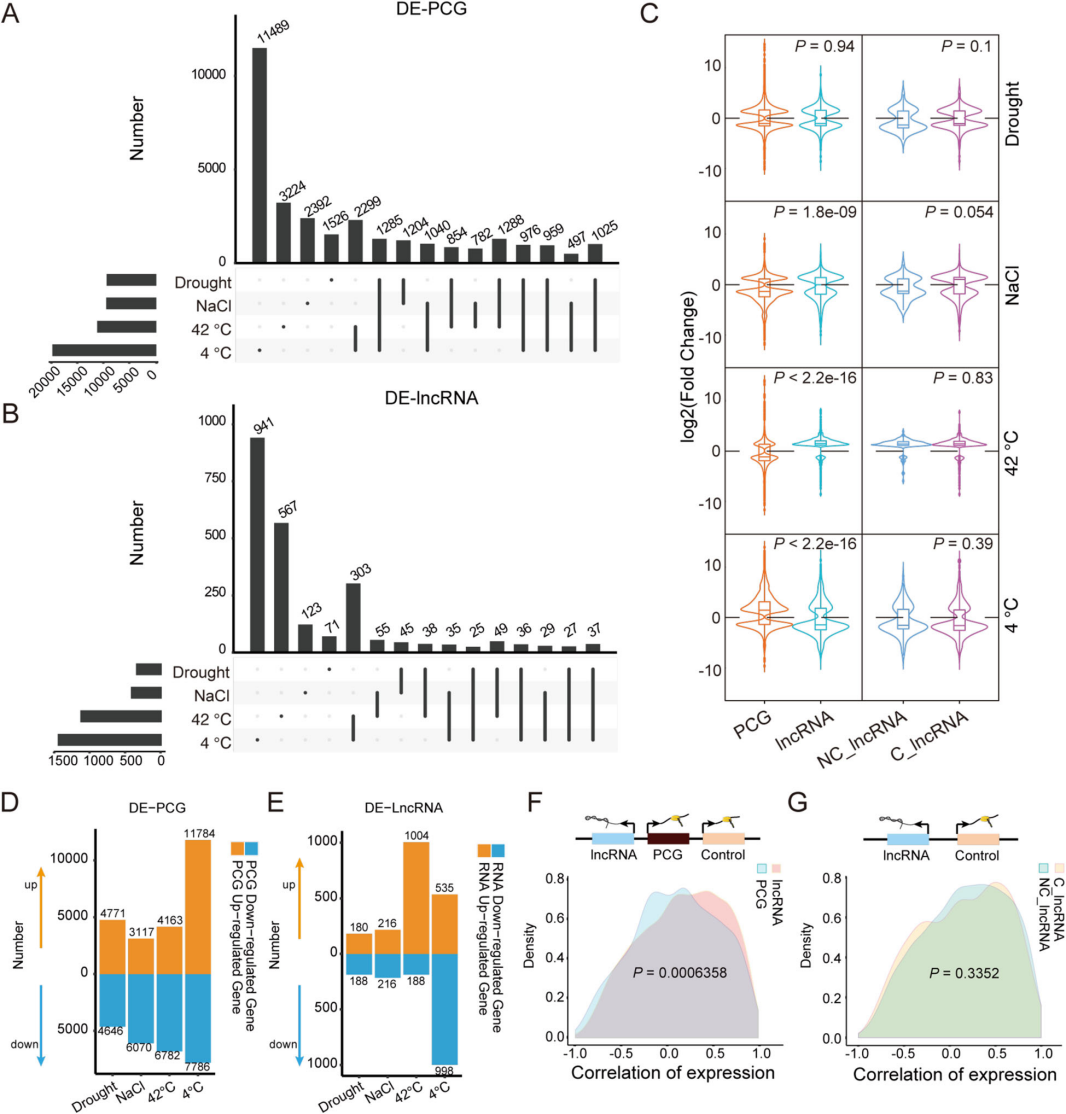
Expression patterns of lncRNAs and PCGs under four abiotic stressors. **A**: Upset plot showing the number of differentially-expressed genes (DE-PCGs) under each stress condition. **B**: Upset plot showing the number of differentially-expressed lncRNAs (DE-lncRNAs) under each stress condition. **C**: Expression amplitude of differentially-expressed PCGs, lncRNAs, conserved (C-)lncRNAs, and non-conserved (NC-)lncRNAs under each stress condition. The *p-*values were calculated by two-sided Wilcoxon signed-rank test. **D**: Bar chart showing the distributions of up-regulated and down-regulated PCGs under stress conditions. **E**: Bar chart showing the distributions of up-regulated and down-regulated lncRNAs under stress conditions. **F**: Density distributions of the Pearson correlation coefficients between adjacent PCG-lncRNA pairs. Each such PCG was also paired with its most adjacent PCG as a control. **G**: Density distributions of the Pearson correlation coefficients between adjacent C-lncRNA-PCG and NC-lncRNA-PCG pairs. The *p-*values were calculated by two-sided Wilcoxon signed-rank test

Plant lncRNAs are reported to be actively involved in the molecular regulation of responses to environmental stimuli and stresses. Abiotic stress is a serious threat that can lead to significant losses of all field crops, including cotton [41]. To validate the association of lncRNA expression with stress in cotton, we performed four abiotic stress assays on cotton seedlings: drought, sodium chloride (NaCl), heat, and cold treatment. The leaves of the treated seedlings were harvested for RNA-seq profiling (Fig. 1), which revealed that both lncRNAs and protein-coding genes (PCGs) tended to be expressed specifically under stress; however, In both lncRNAs (total:8,514) and PCGs(total:70,478), the percentage of differentially expressed lncRNAs (DE-lncRNAs)was lower than the percentage of differentially expressed protein coding genes (DE-PCGs) (Fig. 2A-B; Table S3). The DE-lncRNAs showed a significant divergence of expression pattern relative to PCGs when plants were subjected to abiotic stresses, especially heat and cold. In particular, high temperatures tended to up-regulate lncRNAs, whereas low temperatures down-regulated them (Fig. 2C-E). Meanwhile, C-lncRNAs and NC-lncRNAs showed no significant difference in terms of their stress-responsive expression patterns (Fig. 2C; Table S3). Finally, biological replicates of the RNA-seq results showed high consistency (Fig S1). These findings suggest that abiotic stresses, and especially temperature stress, proactively stimulate lncRNA expression more than PCG expression. The correlation between lncRNAs and PCGs in response to stimuli has yet to be studied.

### Co-expression of lncRNAs with adjacent PCGs

The lncRNA-mediated regulation of gene expression occurs in either *cis* or *trans* [42]. To investigate potential patterns in the mode by which lncRNAs regulate PCGs, we examined the association of their expression profiles in the context of abiotic stress. Here we defined a 1:1 orthologous adjacent PCG that co-expressed with a lncRNA within 50 kb as being subject to a *cis* effect of the lncRNA, consistent with the latest literature [5, 32]. The correlation of expression between lncRNAs and their adjacent PCGs (lncRNA/Control) was significantly high (Fig. 2F; *p* = .0006358, Table S4) compared with that between neighboring PCGs (PCG/Control). And no significant difference was found between C-lncRNA/Control and NC-lncRNA/Control (Fig. 2G; *p* = .3352, Table S5). The above results are consistent with documented lncRNA dynamics in mice [5], and indicate that most lncRNAs tend to play a role in *cis* gene regulation. By combining the expression pattern in stress and expression with adjacent PCGs, we also found that lncRNA conservation level is independent of its function and regulation pattern.

### Functional examination of lncRNAs in allotetraploid cotton

**Fig. 3:**
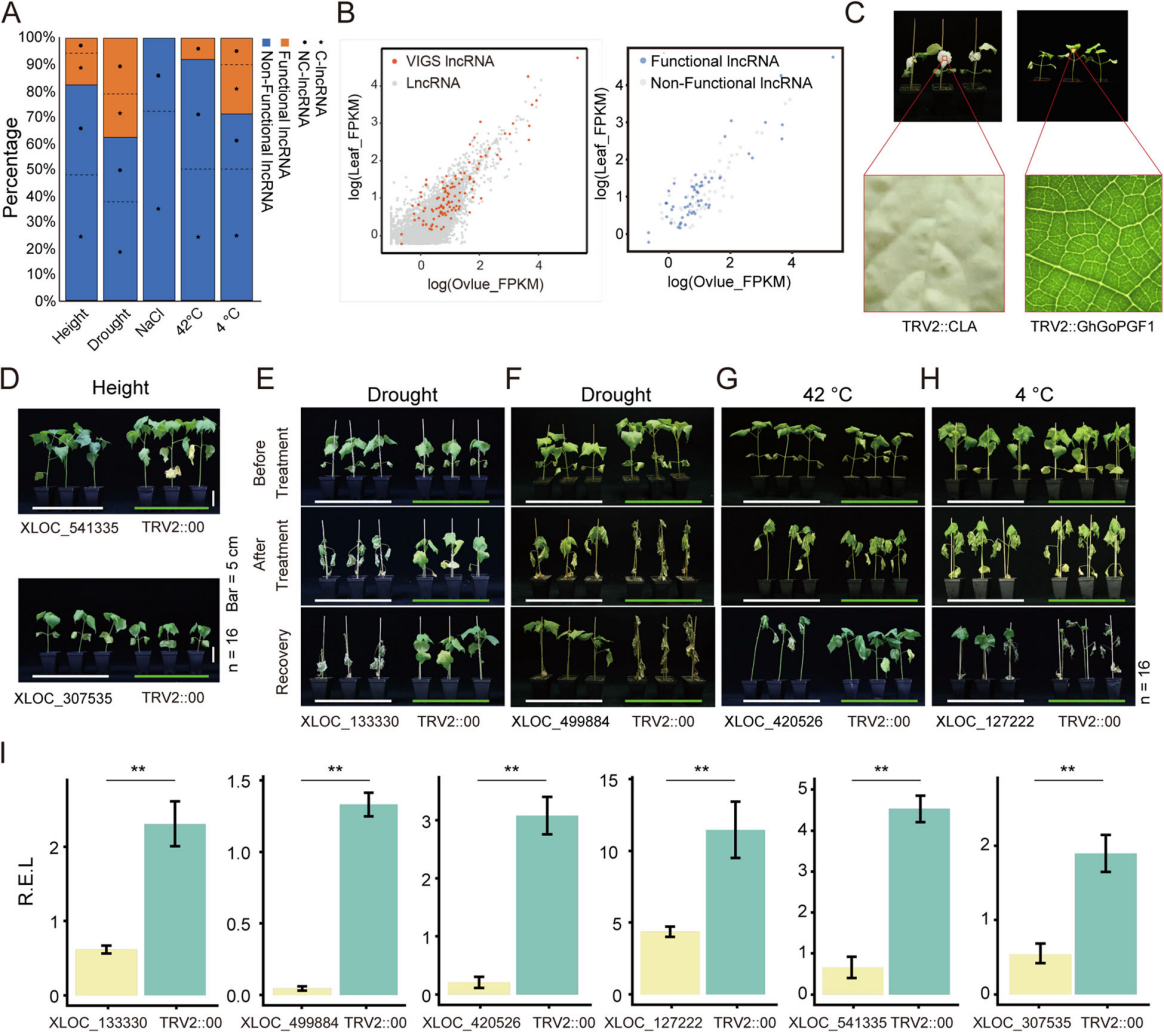
Results of the low-throughput VIGS screening for functional lncRNAs in *G. hirsutum*. **A**: Bar chart showing the numbers of functional and non-functional lncRNAs that exhibited differences in the low-throughput VIGS screening. **B**: The dot plot on the left shows the lncRNA expression distribution in leaves and ovules. Red dots: lncRNA candidates in the preselection for VIGS. Grey dots: all other lncRNAs. The dot plots on the right show the respective expression distributions of functional and non-functional lncRNAs in leaves and ovules. Blue dots: functional lncRNAs. Grey dots: non-functional lncRNAs. **C**: VIGS positive controls, TRV2::*CLA* and TRV2::*GhGoPGF1. ***D**: Photo of VIGS-treated seedlings (*n* = 16) for two lncRNAs potentially affecting plant height. **E-H**: Photos of VIGS-treated seedlings (*n* = 16) for four lncRNAs potentially involved in abiotic stress response. AT, after stress treatment; BT, before stress treatment; RE, after recovery from stress treatment. **I**: Histogram showing relative expression of lncRNAs compared with the TRV2 vector control after VIGS. Data are shown as the means and SDs of three biological replicates. Two asterisks indicate significant difference between samples (Student’s *t*-test, **, *p* < .01)

Several functional studies have reported that lncRNAs can play roles in growth, development, and abiotic stresses in rice, cotton, and other plants [24, 28, 43–47]. Therefore, in this study, we carried out a functional evaluation of lncRNAs in upland cotton (*G. hirsutum*, acc. Texas Marker-1 [TM-1]) by applying virus-induced gene silencing (VIGS) treatment and assessing plant height and tolerance of four abiotic stresses (drought, NaCl, heat [42 ℃], and cold [4 ℃]) (Figs. 1 and 3A). We also evaluated transcriptional activity in leaves in terms of FPKM. Candidate lncRNAs were selected on account of having top-ranking transcriptional activity. Ultimately, 67 NC-lncRNAs and 44 C-lncRNAs were cloned and subjected to VIGS. And 56 (out of 111) lncRNAs affected the phenotype, suggesting they are protentionally involved with correlated biological functions (Figs. 1 and 3B). In terms of lncRNA function, 20 (out of 111 observed) affected plant height, and 34 (out of 90 observed) affected plant death rate in drought abiotic stress. Both positive and negative correlation between the lncRNA and the phenotype were observed as shown in Table S6. Zero lncRNAs (out of 36 observed) were found to be related to NaCl tolerance. One (out of 12 observed) was related to heat tolerance, and five (out of 13 observed) showed differential response to cold stress (Fig. 3A, Table S6). Overall, most of the potential functional lncRNAs that were screened were associated with abiotic stress.

The selection of phenotypic lines after VIGS is shown in Fig. 3C-H, with transcription suppression of the corresponding lncRNA shown in Fig. 3I. The primary results identified *XLOC_133330* and *XLOC_499884* as related to drought stress, *XLOC_420526* and *XLOC_127222* as associated with temperature changes, and *XLOC_541335* and *XLOC_707056* as involved in plant development regulation. Detailed information and phenotypic observations of lncRNAs after VIGS silencing are given in Table S6. These findings indicate that cotton lncRNAs are actively associated with growth and stress tolerance regulation.

### Functional validation of lncRNAs identified in the low-throughput screen

To validate the accuracy of the low-throughput functional examination, representative functional lncRNAs were further assessed. Specifically, we examined two candidates from the functional pilot tests to double-check their phenotypes and examine their potential regulation patterns (*cis*/*trans*).

**Fig. 4:**
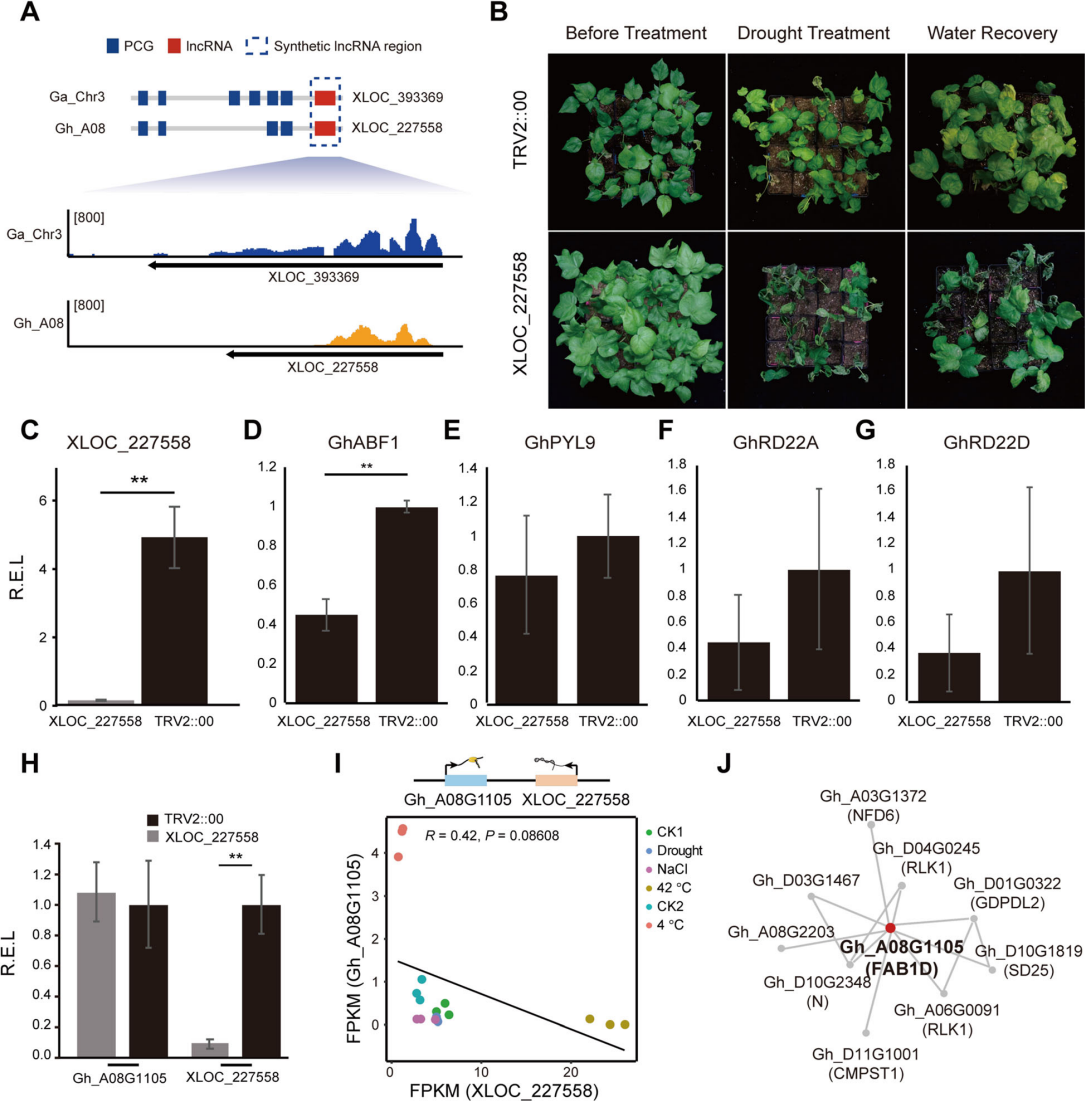
A representative C-lncRNA that screened as affecting cotton seedling drought tolerance. **A**: Schematic showing the collinear position of *XLOC_227558*, between *Gossypium arboreum* (Ga) chr3 and *Gossypium hirsutum* (Gh) A08. This gene is syntenic with *XLOC_227558*. The lower graph shows a stack view of the locus with leaf RNA-seq reads from Ga and Gh. Blue box, PCGs; red box, lncRNAs; dashed box, syntenic lncRNA region. **B**: Photos showing cotton seedlings (*n* = 16) treated with TRV2::*XLOC_227558* and TRV2::00 before drought treatment, after drought treatment, and after water recovery. Bar = 5 cm. **C**: Histogram showing the relative expression level of *XLOC_227558* in TRV2::*XLOC_227558* and TRV2::00 treated plants as quantified by qRT-PCR. Error bar: standard error of the mean. Asterisks indicate significant difference between samples (Student’s *t*-test, *, *p* < .05; **, *p* < .01). R.E.L., relative expression level. **D-G**: Histograms showing the respective relative expression levels of *GhABF1*, *GhPYL9*, *GhRD22A*, and *GhRD22D* in TRV2::*XLOC_227558* and TRV2::00 treated plants as quantified by qRT-PCR. Error bar: standard error of the mean. Asterisks indicate significant difference between samples (Student’s *t*-test, *, *p* < .05; **, *p* < .01). R.E.L., relative expression level. **H**: Histogram showing the relative expression of *XLOC_227558* and its adjacent PCG *Gh_A08G1105* as determined by qRT-PCR. Error bar: standard error of the mean. Asterisks indicate significant difference between samples (Student’s *t*-test, *, *p* < .05; **, *p* < .01). **I**: Scatterplot showing the linear relationship between expression of *XLOC_227558* and that of the adjacent PCG, *Gh_A08G1105*, under different abiotic stressors. J: Co-expression network of *Gh_A08G1105*

The first, *XLOC_227558*, was a C-lncRNA in *G. hirsutum* on chromosome A08, with a syntenic lncRNA, *XLOC_393369*, in *G. arboreum* on chromosome 3 (Fig. 4A). After silencing *XLOC_227558*, TM-1 seedlings exhibited a drought-sensitive phenotype (Fig. 4B and C, Table S6). ABA plays an important role in plant drought stress response, and some specific genes in the ABA pathway have been singled out as involved, such as *ABSCISIC ACID RESPONSIVE ELEMENT-BINDING FACTOR 1* (*ABF1*), *PYRABACTIN RESISTANCE 1-LIKE 9* (*PYL9*), and *DEHYDRATION-RESPONSIVE PROTEIN RD22* (*RD22*) [48]. To observe whether silencing of *XLOC_227558* affected the ABA pathway, we carried out a quantitative analysis of *ABF1*, *PYL9*, and *RD22*. Expression of *ABF1* was decreased in *XLOC_227558*-silenced plants (Fig. 4D-G). Meanwhile, *XLOC_227558* expression did not correlate with that of its adjacent PCG (*Gh_A08G1105*) (Fig. 4I), and the co-expression network of *Gh_A08G1105* displayed no direct evidence of correlation with drought stress (Fig. 4 F; Table S7).

**Fig. 5:**
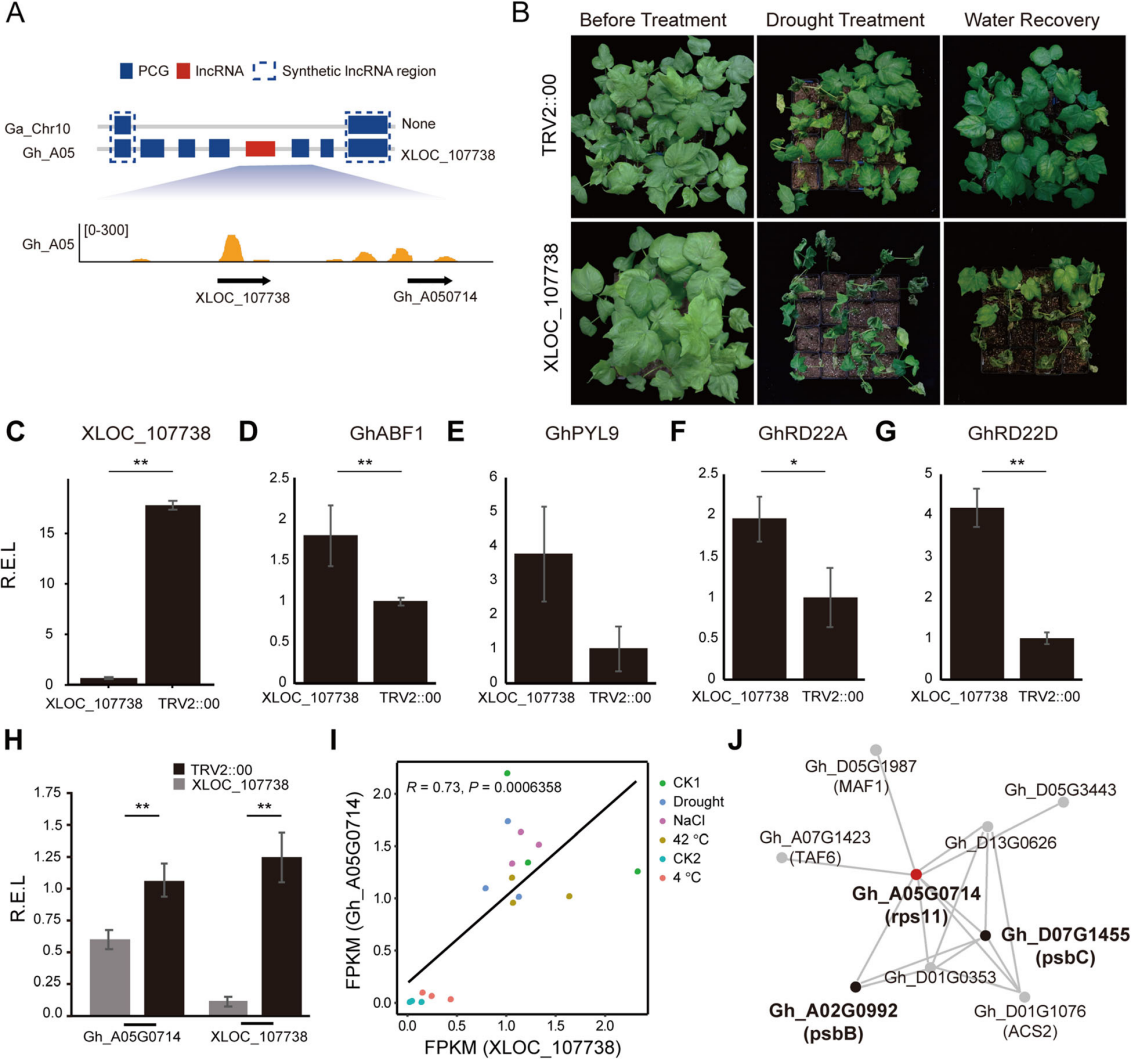
A representative NC-lncRNA may regulate its adjacent gene in *cis* in plants under drought stress. **A**: Schematic showing the collinear position of *XLOC_107738* between *Gossypium arboreum* (Ga) chr10 and *Gossypium hirsutum* (Gh) A05. The lower graph shows a stack view of the locus in Gh. Blue box, PCGs; red box, lncRNAs; dashed box, syntenic PCG region. **B**: Photo showing the phenotype of TRV2::*XLOC_107738* and TRV2::00 treated plants (*n* = 16) before drought treatment, after drought treatment, and after water recovery. Bar = 5 cm. **C**: Histogram showing the relative expression level of *XLOC_107738* in TRV2::*XLOC_107738* and TRV2::00 treated plants as quantified by qRT-PCR. Error bar: standard error of the mean. Asterisks indicate significant difference between samples (Student’s *t*-test, *, *p* < .05; **, *p* < .01). R.E.L., relative expression level. **D-G**: Histograms showing the respective relative expression levels of *GhABF1*, *GhPYL9*, *GhRD22A*, and *GhRD22D* in TRV2::*XLOC_107738* and TRV2::00 treated plants as quantified by qRT-PCR. Error bar: standard error of the mean. Asterisks indicate significant difference between samples (Student’s *t*-test, *, *p* < .05; **, *p* < .01). R.E.L., relative expression level. **H**: Histogram showing the relative expression of *XLOC_107738* and its adjacent PCG *Gh_A05G0714* as determined by qRT-PCR. Error bar: standard error of the mean. Asterisks indicate significant difference between samples (Student’s *t*-test, *, *p* < .05; **, *p* < .01). **I**: Scatterplot showing the linear relationship between expression of *XLOC_107738* and that of the adjacent PCG, *Gh_A05G0714*, under different abiotic stressors. **J**: Co-expression network of *Gh_A05G0714*

The second candidate selected for validation, the NC-lncRNA *XLOC_107738*, also yielded a drought-sensitive phenotype after silencing (Fig. 5A to C, Table S6). In *XLOC_107738*-silenced plants, expression of *ABF1* was likewise decreased while that of *RD22A* and *RD22D* was increased (Fig. 5D-G). Expression of *XLOC_107738* was correlated with that of its adjacent gene *Gh_A05G0714* (*RPS11*). Two genes co-expressed with *Gh_A05G0714* related to photosystem II (Fig. 5 H-J; Table S8), namely *Gh_D07G1455*, which encodes the photosystem II reaction center protein C (psbC), and *Gh_A02G0992*, which encodes the photosystem II reaction center protein B (psbB). As chloroplasts and photosynthesis are actively involved with abiotic and biotic stress in plants [49], we speculated that *XLOC_107738* might act in *cis* to adjust the drought tolerance of cotton. These findings confirmed that both conserved and non-conserved lncRNAs can play a role in cotton stress regulation.

## Discussion

### The rapid evolution of lncRNAs to obtain new functions

Rapid evolution of lncRNAs is commonly observed in both plant and animal kingdoms in the form of interspecies polymorphisms and epigenomic modifications [39, 50]. For example, diverged *Arabidopsis* ecotypes show polymorphisms in the promoter region of the flowering gene *FRIGIDA INTERACTING PROTEIN 1* (*FIP1*), and DNA demethylation associated with lncRNA transcripts can be inherited [51]. Furthermore, whole-genome duplication and domestication can specifically induce lncRNA origin to drive their fast evolution [31]. In comparisons between wild and cultivated cotton varieties, lncRNA transcripts were relatively stable and fixed after polyploidization in wild populations, races, and cultivars alike [31]. These data indicate that environmental stimuli introduced transcriptional variation via inheritable epigenetic modifications, from which beneficial phenotypes were obtained [51–56]. Our study on functional lncRNAs in allotetraploid cotton confirms that the rapid evolution of lncRNAs introduces new, functional non-coding genes.

Among lncRNAs, no statistical difference in the response to stimuli was observed for C-lncRNAs compared with either all lncRNAs or NC-lncRNAs. Furthermore, our primary functional screening indicated that in the cotton lineage, both C-lncRNAs and NC-lncRNAs have biological roles. In our study, the definition of conserved lncRNA is based on sequence similarity; however, conserved lncRNAs reported to be functional show low sequence similarity. Some structural elements, such as an RNA-loop, could be sufficient for lncRNA function; thus, our definition of conservation may underestimate the impacts from features such as short motifs or secondary structures. However, the trend observed in our study also supports that NC-lncRNAs have the chance to gain a new regulatory role and, for cotton, were selected in the cultivated population. These results are in agreement with the notion that epigenetic modifications associated with beneficial traits are under positive selection. We therefore speculate that inheritable and functional epigenetic modifications can play a role in the rise of domesticated traits. Polymorphisms of epigenetic modifications in a natural population may thus be an unknown reservoir of genetic markers for the development of new germplasms. 

### Using the RNA silencing technology of VIGS to validate the LncRNA function

LncRNAs are abundantly present and transcribed in the genome [57]; however their gene structure and transcriptional activity are relatively unstable compared with PCGs [58, 59]. Utilization of cell lines may aid in achieving high-throughput screening with strong transcriptional signal, but lncRNA function is often cell-type specific, thus the cultured cells may not reflect the real functions of the lncRNAs [60]. Multiple published studies have carried out functional lncRNA screenings with various strategies, including reverse genetics, which uses CRISPRi technology [29, 60]; comparative genomics and transcriptome-based prediction across species, wild species, and cultivars [4, 39]; and mapping based on epigenetic recombination lines [54, 61]. High-throughput CRISPRi operations made great improvement on testing the lncRNA function. The typical CRISPRi operation is targeted for short DNA fragment editing of 1 to 10 base pairs, which may not significantly influence RNA transcription at the edited site [62]. Likewise, RNA silencing technology of VIGS used in this study should be an efficient gene operation of functionla lncRNA screening.

In general, most lncRNAs are responsive to or play roles in responses to environmental stimuli. However, it is noteworthy that in both animal and plant populations, high-throughput screening has not identified a specific biological function for C-lncRNAs. The reported low conservation of lncRNAs across species and the screening-out of conserved lncRNAs among functional candidates in this study suggest that the potential functionality of each conserved non-coding gene needs to be considered as an individual case. That is, degree of lncRNA conservation shows little correlation to function at the genome-wide level. This observation favors our conclusion that lncRNA function is unique to species and to lineage over the course of evolution. The results of the present study might be skewed due to the small number of conserved lncRNAs, but that the functional lncRNA profile is unique to each organism is in agreement with most of the reported biological impacts of lncRNAs.

## Conclusions

The impact of lncRNA conservation on expression patterns and functions may operate at the level of individual genes rather than genome-wide. The development of inheritable and functional lncRNAs over evolution can participate in the emergence of adaptive traits.

## Methods

### Plant materials

Plants of the upland cotton (*G. hirsutum*) genetic standard line Texas Marker-1 (TM-1) [63] were obtained from the Agricultural Research Service, U.S. Department of Agriculture, and the Texas Agricultural Experiment Station. Plants were cultivated in a growth chamber at 25 to 28 ℃ with a light/dark cycle of 16/8 hours. All were planted in 6.5 cm * 6.5 cm plastic pots filled with a 1:1 (v/v) mixture of commercial humus:commercial vermiculite.

### Plasmids and constructs

Unique 200- to 400-bp fragments of lncRNAs were amplified from TM-1 cDNA by polymerase chain reaction (PCR) using Ex Taq (Takara, Code No.: RR01AM). The primer list is given in Table S9. PCR products were cloned into E*coR* I-B*amH* I-digested pTRV2 to produce a VIGS vector. Resulting constructs were introduced into *Agrobacterium tumefaciens* strain GV3101 by liquid nitrogen transformation.

### Virus-induced gene silencing

TM-1 plants were cultivated in a growth chamber under conditions of 16 h/8 h light/dark and 21 °C ± 1 °C. Plants used for gene silencing were approximately eight days old when their cotyledons expanded. For each experiment, *A. tumefaciens* harboring pTRV1, pTRV2 (TRV2:00), TRV2::*CLA* (*Cloroplastos alterados 1*) [64], TRV2::*GhGoPGF1* [65], and pTRV2 containing host target genes were grown on Luria broth (LB) agar plates supplemented with 50 µg/mL of kanamycin and 25 µg/mL of rifampicin. The plates were incubated at 28 °C for two days. Plasmids TRV2::*CLA* and TRV2::*GhGoPGF1* were utilized as positive controls, and the empty vector TRV2::00 was the negative control. Silencing of *CLA* causes the plants to become photobleached, and this was used as a silencing efficiency control. For each strain, 3-mL primary liquid culture of LB was inoculated with the above-mentioned antibiotics and was incubated with shaking at 200 rpm at 28 °C for 14 to 16 h. A 1:100 dilution of the primary culture was then inoculated into a secondary liquid induction media culture with 50 µg/mL of kanamycin and 25 µg/mL of rifampicin, which was incubated with shaking at 28 °C for 14 to 16 h at 200 rpm until the OD_600_ was 1.5 to 2.0. The cells were then harvested by centrifugation for ten minutes at 4000 x *g* and were resuspended in a culture medium with 10 mM magnesium chloride, 10 mM MES, and 200 µM acetylacetone. For each culture, a bacterial suspension with an OD_600_ of 2.0 was prepared and incubated in the dark at 28 °C for three to five hours. Cultures containing the pTRV1 vector and the pTRV2 vector with the gene of interest were mixed at a 1:1 ratio. The bacterial suspensions were infiltrated into the cotyledons of the seedlings, and the plants were kept at 21 °C ± 1 °C in a growth chamber with a 16-hour day length and 50 % relative humidity for at least three weeks before use in assays.

### Height observation and stress treatment of cotton plants with virus-induced gene silencing

TM-1 plant height (from cotyledon to growth point) and leaf number were measured about 30 days after VIGS. For drought stress, plants were irrigated with sufficient water, then subject to water restriction until those infected with TRV2::lncRNA or TRV2::00 were dying. Soil water content (SWC) was determined according to the formula: SWC (%) = (Ww − Wd)/(Wd − Wt) × 100 %, where Ww is the wet mass of soil in tube, Wd the dry mass of soil in tube (after over-drying at 80 °C until a constant mass was obtained), and Wt is the mass of the empty tube [24]. Leaf samples were harvested right before water restriction and were immediately frozen in liquid nitrogen and stored at − 80 °C. Plants were photographed before and after the water restriction and subsequent rewatering. For NaCl stress, plants were irrigated with 200 mM NaCl instead of water. For heat stress, the plants were placed in a 42 ℃ incubator until those treated with TRV2::lncRNA or TRV2::00 were dying. For cold stress, plants were placed in a 4 ℃ incubator until those treated with TRV2::lncRNA or TRV2::00 were dying. Survival rates were scored after plants were restored to normal growth conditions for seven days. Each group consisted of 16 replicates.

### cDNA synthesis and quantitative polymerase chain reaction

Total RNA was isolated using an RNA extraction kit (RK-16, Zhong Ding, Nanjing, China), and first-strand cDNA synthesis was performed using HiScript® II (Vazyme). Gene-specific primers were used for the SYBR Green-based qPCR, which was performed on an ABI StepOnePlus system with 20 µL of 100 ng of cDNA, 4 pM of each primer, and 10 µL of AceQ qPCR SYBR Green Master Mix (Vazyme, Nanjing, China) according to the manufacturer’s protocol. The thermal cycle conditions were as follows: 95 °C for three minutes, then 40 cycles of 95 °C for 15 s, 60 °C for 15 s, and 72 °C for 30 s. Relative expression levels were calculated using the 2-ΔΔCt method. *Histone 3* (AF024716) was used as an internal control for normalization. The primer sequences are listed in Table S10.

### Classification of C-lncRNA and NC-lncRNA

The lncRNA sequences and genome coordinate files of *G. arboreum* (Ga), *G. raimondii* (Gr), and *G. hirsutum* (Gh) were previously generated by our laboratory and are deposited in Github repositories (https://github.com/epi-cotton/LncRNA-in-polyploid-cotton). Conservation was determined based on synteny and sequence similarity. MCScanX [66] was used to analyze the multicollinearity between Ga and GhA_T_ and Gr and GhD_T_ (blastn -evalue 10^^−10^ -max_target_seqs 1; MCscanX file -b 2, -s 5) [31]. Individual transcript expression levels were quantified in terms of the fragments per kilobase of exon per million fragments (FPKM) by StringTie [67].

### Expression profiling of stress-treated cotton plants by RNA-seq

Cotton seedlings with four expanded leaves were used for stress treatments. Cold treatment was carried out at 4 ℃, heat at 42 ℃, salt with 200 mmol NaCl, and drought in the form of water restriction. All plants were grown in chambers with a light/dark cycle of 16/8 hours. Leaf tissues were harvested after 30 days of treatment and frozen with liquid nitrogen for RNA extraction and sequencing (Table S11). Read quality was assessed before and after trimming using FastQC [68]. Reads were aligned to the reference genome *G. hirsutum* V1.0 [36] using HISAT2 [69], and the resulting BAM files were sorted and indexed using SAMtools [70]. Expression (FPKM) was calculated by StringTie [67], and differential gene expression was determined using DESeq [71] with a false discovery rate threshold of 0.05.

### Co-expression with adjacent coding genes

Cotton lncRNAs (with filtered lncRNAs) were assigned to their nearest PCG using bedtools based on the distance between gene bodies. Each PCG assigned to a lncRNA was then matched to its immediate neighboring PCG, which was used as a control. We estimated the Pearson’s expression correlation and performed a two-sided Wilcoxon signed-rank test between the lncRNA/control and PCG/control pairs using.The same analysis was also carried out for C-lncRNA/control and NC-lncRNA/control pairs.

### Co-expression network

The co-expression network for lncRNAs was constructed as described previously. Positive and negative co-expression networks for PCGs were generated online (http://structuralbiology.cau.edu.cn/gossypium/) [72].

### General statistics and plots

All statistical analyses were performed in R (https://www.r-project.org/) using the packages *data.table* and *stats*. All plots were generated in R using the packages *ggplot2* (https://cran.r-project.org/web/packages/ggplot2/index.html), *ggpubr* (https://cran.r-project.org/web/packages/ggpubr/index.html), *pheatmap* (https://cran.r-project.org/web/packages/pheatmap/index.html), and *gmodels* (https://cran.r-project.org/web/packages/gmodels/index.html).

## Supplementary Information

**Additional file 1: Figure S1.** Examination of repeatability among RNA sequencing samples. A: Heatmaps correlating expression levels among samples based on the RNA-seq profiles of cotton seedlings treated with drought, NaCl, and heat. B: Heatmaps correlating expression levels among samples based on the RNA-seq profiles of cotton seedlings treated with cold.

**Additional file 2: Figure S2.** Examination of PCG and lncRNA associations. A: Phenotypes of seedlings treated with positive control TRV2::*CLA*. B: Phenotypes of seedlings treated with TRV2::00 and positive control TRV2::*GhGoPGF1*. C: Soil water content before drought treatment for seedlings receiving TRV2::*XLOC_227558*and TRV2::00. D: Soil water content after drought treatment for seedlings receiving TRV2::*XLOC_227558* and TRV2::00. E: Soil water content before drought treatment for seedlings receiving TRV2:: *XLOC_107738* and TRV2::00. F: Soil water content after drought treatment for seedlings receiving TRV2:: *XLOC_107738* and TRV2::00.

**Additional file 3: Table S1.** List of C-lncRNAs in GhA_T_ and corresponding syntenic lncRNA in Ga.

**Additional file 4: Table S2. **List of C-lncRNAs in GhD_T_ and corresponding syntenic lncRNA in Gr.

**Additional file 5: Table S3. **Counts of differentially-expressed PCGs and lncRNAs under four stress treatments.

**Additional file 6: Table S4. **Expression of adjacent PCG-lncRNA pairs and adjacent PCG- PCG pairs.

**Additional file 7: Table S5. **Expression of adjacent C-lncRNA-PCG and NC-lncRNA-PCG pairs.

**Additional file 8: Table S6.** Expression, conservation level, and associated phenotype of lncRNAs subjected to VIGS.

**Additional file 9: Table S7. **Annotations of genes co-expressed with *Gh_A08G1105*.

**Additional file 10: Table S8.** Annotations of genes co-expressed with *Gh_A05G0714*.

**Additional file 11: Table S9. **The primers for lncRNA VIGS construct cloning.

**Additional file 12: Table S10.** qPCR primers used in this study.

**Additional file 13: Table S11.** Statistics of RNA-seq libraries in this study.

## Data Availability

The lncRNA sequences and genome coordinate files are provided at https://github.com/epi-cotton/LncRNA-in-polyploid-cotton. The Illumina RNA-seq data are available at the Sequence Read Archive under the accession number PRJNA631851.

## Abbreviations

lncRNA: Long non-coding RNAs (lncRNAs)
C-lncRNA: Conserved lncRNA
NC-lncRNA: Non-conserved lncRNA
PCG: Protein-coding gene
FPKM: Fragments Per Kilobase of exon model per Million mapped fragment
DE-PCGs: Differentially expressed protein-coding genes
DE-lncRNAs: Differentially expressed lncRNAs
kb: Kilobase
bp: Base pair
TM-1: Texas Marker-1
