# Supplementary figures and images for: Functional examination of lncRNAs in allotetraploid Gossypium hirsutum
Source: BMC Genomics. 2021 Jun 13;22:443. doi: 10.1186/s12864-021-07771-3 (PMC8201905; doi:10.1186/s12864-021-07771-3)

A

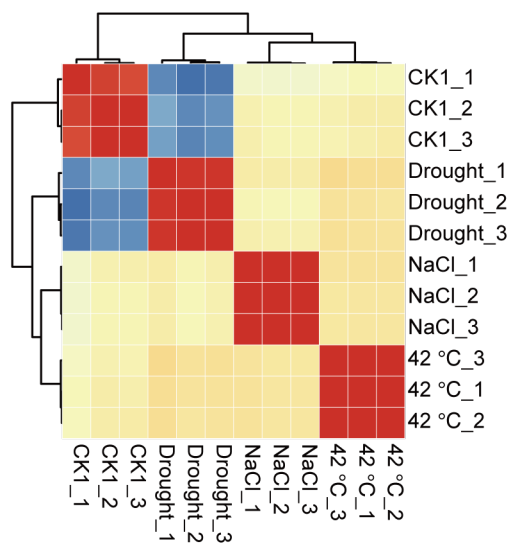

B

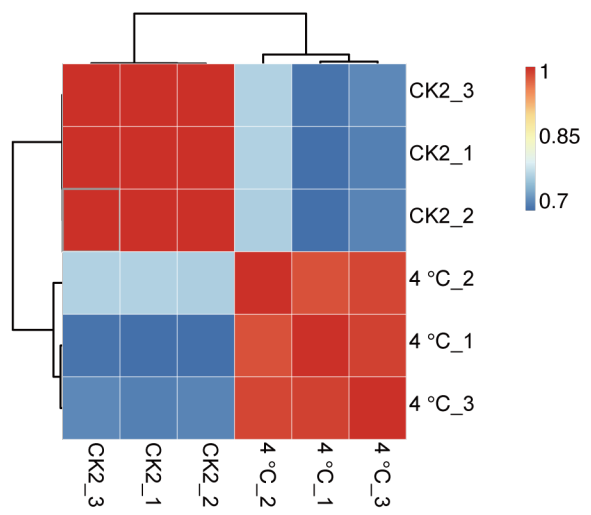

Supplement: Supplementary file 1 — Additional file 1: Figure S1. Examination of repeatability among RNA sequencing samples. A: Heatmaps correlating expression levels among samples based on the RNA-seq profiles of cotton seedlings treated with drought, NaCl, and heat. B: Heatmaps correlating expression levels among samples based on the RNA-seq profiles of cotton seedlings treated with cold. [file 12864_2021_7771_MOESM1_ESM.pdf]

**A**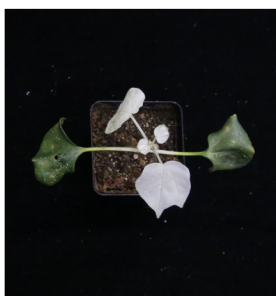

TRV2::CLA

**B**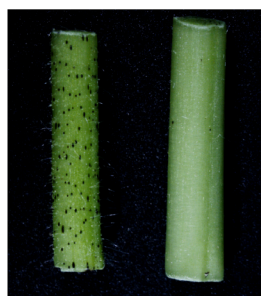

TRV2::00 TRV2::GhGoPGF1

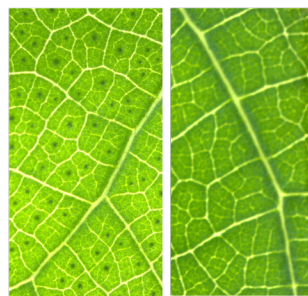

TRV2::00 TRV2::GhGoPGF1

**C**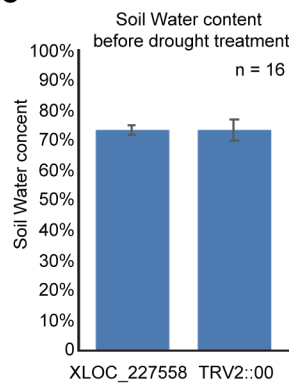**D**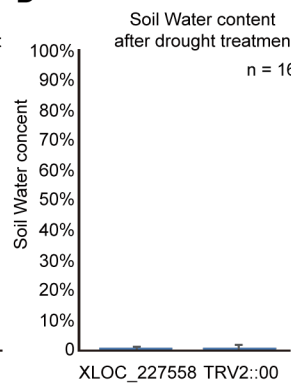**E**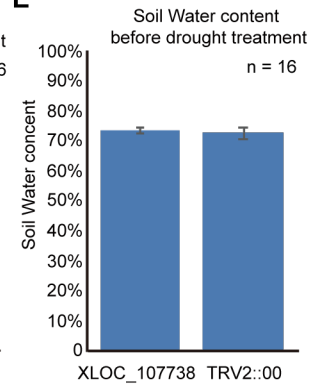**F**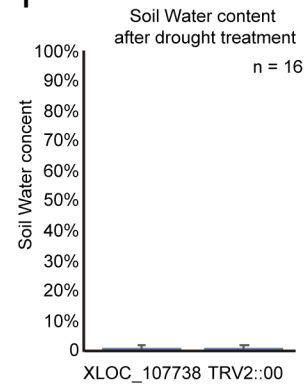

Supplement: Supplementary file 2 — Additional file 2: Figure S2. Examination of PCG and lncRNA associations. A: Phenotypes of seedlings treated with positive control TRV2::CLA. B: Phenotypes of seedlings treated with TRV2::00 and positive control TRV2::GhGoPGF1. C: Soil water content before drought treatment for seedlings receiving TRV2::XLOC_227558and TRV2::00. D: Soil water content after drought treatment for seedlings receiving TRV2::XLOC_227558 and TRV2::00. E: Soil water content before drought treatment for seedlings receiving TRV2:: XLOC_107738 and TRV2::00. F: Soil water content after drought treatment for seedlings receiving TRV2:: XLOC_107738 and TRV2::00. [file 12864_2021_7771_MOESM2_ESM.pdf]
